# Venereal Diseases

**Published:** 1898-04

**Authors:** 


VENEREAL DISEASES have been radically healed by Homœopathy much more surely, with less trouble, and without any sequelæ; for without disturbing or destroying the local manifestation it heals the internal fundamental disease from within only.—(Hahnemann in *Chronic Diseases*, new ed.)

